# Toward accurate prediction of pediatric epidemic disease patient volume in the Chaoshan region: A deep learning framework

**DOI:** 10.1016/j.isci.2026.115211

**Published:** 2026-03-03

**Authors:** Siqi Wang, Jinlian Fang, Yulin Chen, Hui Chen, Yaowen Chen, Yangxin Ye, Shixin Lai, Xiaolei Zhang, Hongwu Wang, Qiuling Tang

**Affiliations:** 1 Department of Pediatrics, Second Affiliated Hospital of Shantou University Medical College, Shantou 515041, China; 2 College of Engineering, Shantou University, Shantou 515063, China; 3 Department of Neurosurgery, Cancer Hospital of Shantou University Medical College, Shantou 515041, China; 4 Department of Biomedical Engineering, Guangzhou Xinhua University, Guangzhou 510520, China; 5 Department of Radiology, Second Affiliated Hospital of Shantou University Medical College, Shantou 515041, China

**Keywords:** Health sciences

## Abstract

Accurate prediction of pediatric epidemic infectious diseases is critical for effective prevention and personalized treatment. Herein, we developed a deep learning framework for the epidemiological characteristics of the Chaoshan region, using electronic health records data from 278,506 pediatric outpatient and emergency visits at the Second Affiliated Hospital of Shantou University Medical College between 2017 and 2023. Our framework is designed to learn pediatric representations that capture local epidemic dynamics and to meet regional clinical prediction needs. Results demonstrate that the framework achieves strong predictive performance on the regional dataset. Our framework yields at least a 6.12% improvement over its counterparts in terms of average correlation coefficient; it achieves the lowest errors in both root-mean-square error (RMSE = 0.130) and mean absolute scaled error (MASE = 0.610). Our framework provided targeted decision support for local healthcare institutions in workforce allocation, medication, and supply planning, thereby contributing to improved prevention strategies.

## Highlights

• LSTM-BEATS learns region-specific pediatric epidemic dynamics from 2017 to 2023 Chaoshan EHR
• LSTM-BEATS with trend seasonality modules and self-attention improves multivariate prediction
• Achieved lowest errors, RMSE 0.130 and MASE 0.610 with higher correlations
• Predictions support proactive staffing and medication planning during influenza-season surges

## Graphical abstract

**Figure:**
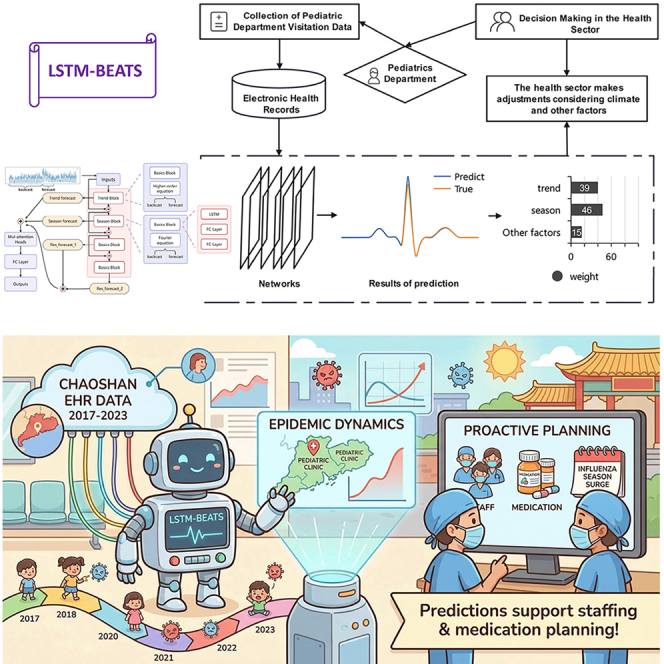

## Teaser

Wang et al. develop a deep learning framework (LSTM-BEATS) that accurately predicts pediatric epidemic patient volume in the Chaoshan region by capturing local epidemiological dynamics. This model outperforms traditional methods and enables proactive resource planning—helping hospitals optimize staffing and medical supply allocation ahead of seasonal outbreaks

## Introduction

Electronic health records (EHRs) refer to the computerized medical records that encapsulate information pertaining to healthcare entities associated with patients. These records encompass an individual’s basic personal information as well as their medical condition, with the primary objective of facilitating the provision of healthcare and related services. The vast array of latent patterns present within EHR is rapidly being harnessed for modeling and decision-making within the healthcare sector.^1^^,^^2^^,^^3^^,^^4^ Children are a key demographic in many nations, facing a crucial developmental stage. Their vulnerability to infections and other factors puts a strain on pediatric departments, impacting resource distribution and pediatricians' workload. This also raises the risk of misdiagnosis and medical oversight. Identifying growth and development patterns in children, influenced by the environment, helps hospitals manage pediatric resources more effectively and ease the pressure on pediatricians. Regular analysis of EHR can further support healthcare departments in dealing with infectious disease surges, such as those seen during flu seasons and other natural outbreaks.

To achieve this goal, numerous researchers have employed various methods for disease onset prediction.^5^ Among these, the most common approach involves utilizing time series models to predict epidemiological behaviors by modeling historical surveillance data. The global issue of overcrowding in emergency departments (EDs) leads to many negative effects, primarily due to the inefficient allocation of medical resources.^6^^,^^7^ Therefore, addressing the issue of hospital resource allocation becomes critical, and methods for effectively predicting the potential number of future patients have garnered attention. Numerous researchers have proposed machine learning models for predicting the likelihood of hospitalization and estimating waiting times.^8^^,^^9^ For instance, studies have used pediatric ED data to predict future visits for asthma,^10^ epilepsy,^11^ and mental health patients.^12^ Collectively, these studies aimed to address resource allocation challenges in hospital pediatric outpatient and ED, thereby reducing the likelihood of misdiagnosis and promoting the healthy development of children.

In previous studies, many researchers have employed traditional machine learning methods to establish time series prediction models for predicting the future occurrence of diseases.^13^^,^^14^^,^^15^ However, additional protocols are required to capture outliers and mutation points to address complex situations.^16^^,^^17^ Nevertheless, the efficiency of traditional machine learning algorithms significantly diminishes when dealing with large volumes of data, indicating a lack of capability for big data analysis.^18^ In recent times, neural networks have achieved high-precision results by mimicking the information transmission effects of human brain neurons, leading to the widespread application of deep learning in the medical field. Researchers have constructed deep learning models to predict the onset of conditions such as epilepsy,^19^ diabetes,^20^ and cardiovascular diseases,^21^ surpassing the accuracy of traditional machine learning methods. To improve the accuracy of predictive outcomes, researchers have explored more ensemble models. Chen and Qian proposed a model combining neural networks (NNs) with factorization machines (FMs) for predicting pediatric sepsis.^22^ This model employed FM to obtain dense latent vectors, accelerating model convergence and fully leveraging the feature representation capabilities of FM. Reddy and Delen adopted a combination of recurrent neural networks (RNNs) and long short-term memory (LSTM) to extract temporal relationships from longitudinal EHR clinical data, predicting the 30-day readmission probability for patients with lupus.^23^

It is evident that deep learning methods hold immense potential in the medical field. This study proposes an LSTM-BEATS model for predicting the number of pediatric epidemic cases in the Chaoshan region. The model is designed to capture local epidemiological characteristics and meet the clinical prediction needs of the region. The LSTM-BEATS model integrates a trend module, a seasonal periodic module, and a residual module, and introduces a multi-head self-attention mechanism. This approach effectively captures data features and detects structural changes and mutation points in historical data. The model utilizes past data from pediatric departments and the ED to predict future pediatric disease trends in children, demonstrating competitive performance. The contributions of this article can be summarized as follows:

1. Analyzed pediatric EHRs from the Second Affiliated Hospital of Shantou University Medical College over the past seven years, established a dataset of high-incidence pediatric diseases with regional climatic characteristics, and predicted the number of pediatric patients. The results offer valuable insights for healthcare policy formulation in similar environments.
2. Development of a universal model capable of capturing both structured features and mutation points.
3. The LSTM-BEATS possesses strong interpretability, allowing for the individual examination of trend variations, seasonal cyclical changes, and other factor changes, which is of significant importance for health organizations in responding to emergent situations such as novel influenza outbreaks.
4. LSTM-BEATS has provided targeted support for local healthcare institutions in human resource allocation, medication planning, and supply management. This capability is particularly significant for local health organizations to respond to emerging public health emergencies, such as novel influenza outbreaks.

## Results

In our study, we considered 21 indicators derived from pediatric EHR of the Second Affiliated Hospital of Shantou University Medical College, the largest children’s medical center in Chaoshan region, including: “Male,” “Female,” “2 years old,” “3 years old,” “4 years old,” “5 years old,” “6 years old,” “7 years old,” “8 years old,” “9 years old,” “10 years old,” “11 years old,” “12 years old,” “13 years old,” “14 years old,” “Respiratory system diseases,” “Neuromuscular system diseases,” “Digestive system diseases,” “Endocrine system diseases,” “Urinary system diseases,” and “Mental and psychological disorders.”

We conducted experiments with various models, evaluating their predictive performance using metrics such as root-mean-square error (RMSE) and mean absolute scaled error (MASE), correlation coefficient, Lag and Peak Error between different features. Moreover, we performed an in-depth analysis of the prediction results, incorporating a newly designed multi-head attention mechanism to distribute weights across different modules, thereby enhancing the interpretability of the model. Several state-of-the-art models were included in the experiments for comparison, further demonstrating the reliability and superiority of our proposed approach.

### ARIMA

The autoregressive integrated moving average model (ARIMA)^24^ is a renowned method for time series prediction, capable of handling non-stationary time series data. Through differencing and other techniques, it renders the data stationary.

### N-BEATS

Neural basis expansion analysis for time series predicting (N-BEATS) is an advanced deep learning approach for time series prediction. Characterized by its flexibility and interpretability, the N-BEATS model can adapt to various time series datasets by altering the quantity and types of basic blocks. A key feature of this model is that its entire architecture consists exclusively of fully connected layers. It is available in two versions: a general model and an interpretable model, with the latter being adept at capturing seasonal and trend characteristics.

### TCN

Temporal convolutional networks (TCNs)^25^ are an advanced deep learning model designed for processing time series data, introduced by Bai, Shaojie et al. in 2018. This model boasts the advantages of parameter sharing and effective capture of long-term dependencies, demonstrating exceptional performance across a variety of tasks.

### Prophet

Prophet is an open-source time series predicting tool developed by Facebook, characterized by its integration of various statistical and machine learning techniques to provide a flexible and accurate approach for predicting time series data. It has the advantages of seasonality adjustments and holiday effects. However, for multivariate time series data, or data with unclear seasonality or trends, and in handling nonlinear data, the predictive performance of Prophet may not be ideal.

### Experimental results

We first predicted 21 indicators for the test set (i.e., the year 2023) using a predefined ratio of past steps to future steps, set at 7:1, as shown in Table 1. The experimental results demonstrate that our model exhibits superior performance. Specifically, LSTM-BEATS’s RMSE is 0.042 lower than the traditional ARIMA model and 0.022 lower than other advanced deep learning models such as TCN. Notably, among the evaluated models in terms of MASE, only LSTM-BEATS is less than 1, achieving 0.610, indicating that our model’s predictive accuracy is better than the benchmark models. Additionally, we calculated the average correlation coefficient, and the correlation between our model’s predictions and actual observations is higher.

**Table 1:** Model performance metrics - RMSE, MASE, and correlation coefficient

Model	RMSE	MASE	Average correlation coefficient	DM (*p* < 0.05)
ARIMA	0.172	1.25	0.089	14.0835
N-BEATS-G	0.145	1.36	0.618	5.885
N-BEATS-I	0.141	1.58	0.637	3.253
LSTM	0.143	0.734	0.504	4.333
TCN	0.152	1.03	0.520	5.516
Prophet	0.214	1.46	0.021	10.967
LSTM-BEATS	0.130	0.610	0.676	/

DM, Diebold-Mariano; MASE, mean absolute scaled error; RMSE, root-mean-square error.

The Diebold-Mariano (DM) test^26^ results indicate that LSTM-BEATS exhibits statistically significant differences in predicting performance compared with all benchmark models (*p* < 0.05). In particular, its superiority is most pronounced relative to ARIMA (DM = 14.08) and Prophet (DM = 10.967). It also significantly outperforms TCN (DM = 5.516) and N-BEATS-G (DM = 5.885). By contrast, when compared with N-BEATS-I (DM = 3.253), the performance difference is relatively small, suggesting comparable predictive accuracy. Overall, these findings further confirm that LSTM-BEATS consistently achieves substantial improvements in prediction performance across all comparisons, demonstrating strong modeling capability and generalization potential.

In addition, we report 95% confidence intervals (CIs) for all metrics in Table 1 (RMSE, MASE, and correlation), both at the individual target level and for their macro-averaged values, as listed in Table S1. To appropriately account for serial dependence in the time series, a block bootstrap with a block length of seven days was applied to the 2023 test period for each series. When reporting macro-averaged results, a second resampling stage was conducted across targets, resulting in a stratified bootstrap procedure.

To obtain a more robust estimate of the generalization error, we evaluate the models using a block bootstrap approach with a window length of seven days. A multi-level stratified resampling scheme is employed to compute macro-averaged performance metrics, with 500 bootstrap replications at both the first and second levels. The results demonstrate that the proposed LSTM-BEATS model consistently outperforms all benchmark methods across all evaluation metrics. In terms of predictive accuracy, LSTM-BEATS achieves the lowest RMSE of 0.1532, with a 95% confidence interval of [0.1425, 0.1737]. Moreover, the confidence intervals of the remaining metrics exhibit little to no overlap with those of the baseline models, further confirming the statistical robustness of the observed performance gains. It is worth noting that the original test set may correspond to a relatively “calm” period or one characterized by a clear trend, whereas the block bootstrap procedure simulates a wide range of more challenging fluctuation patterns. Consequently, compared with the RMSE obtained from a single train-test split (0.160), the block bootstrap yields a more conservative error estimate, reflecting the model’s average predictive performance under diverse historical resampling scenarios.

**Figure 1:**
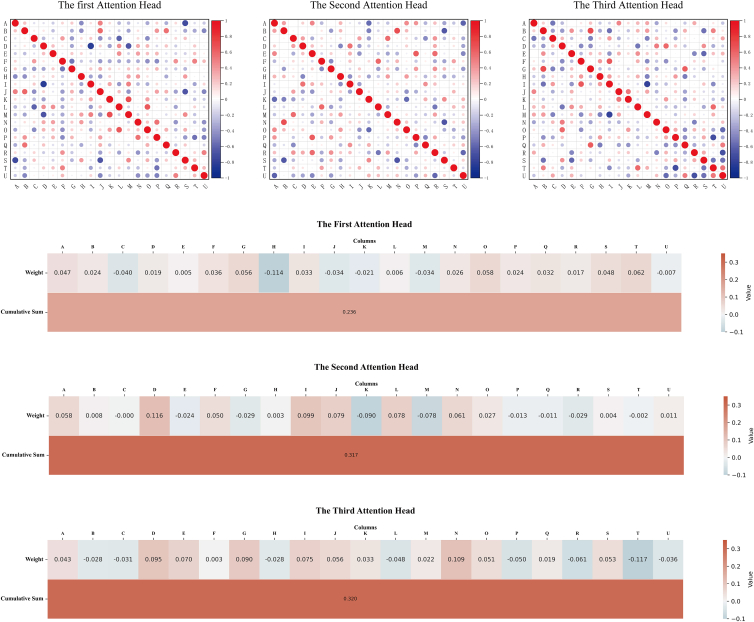
Distribution of weights across different modules in multi-head attention mechanism The three subplots of the first row illustrate the correlations between features in different attention heads, while the remaining three subplots provide a more intuitive view of the degree of influence of different attention heads on the results. A darker color indicates a higher attention score and a higher positive correlation score.

To provide a more intuitive explanation of LSTM-BEATS, we present the weights of the multi-head attention mechanism in the form of a heatmap (Figure 1). This is one of the mainstream interpretability techniques, namely Saliency-based techniques. Examining the influence of each module and feature on the outcome helps to understand the importance of the trend module, the seasonal periodic module, and the residual module in the prediction results. Within the multi-head attention mechanism, we observe the weights of the three heads after the completion of training. The first head inputs the trend module, the second head inputs the seasonal periodic module, and the third head inputs the residual module for the prediction of 21 features. Among the features predicted, each feature plays a different role in the output weights for other features. Each element (*i*, *j*) represents the model’s attention to the element at the *j*^th^ position in the sequence when generating the output at the *i*^th^ position. A higher score at (*i*, *j*) indicates that the element at the *j*^th^ position has a greater influence when generating the *i*^th^ element. According to the weight heatmap, we can observe that the model’s attention to each module is distinct, which is beneficial for understanding the impact of multiple factors on the outcome in real-world business scenarios and can assist in addressing factors with a stronger influence.

**Figure 2:**
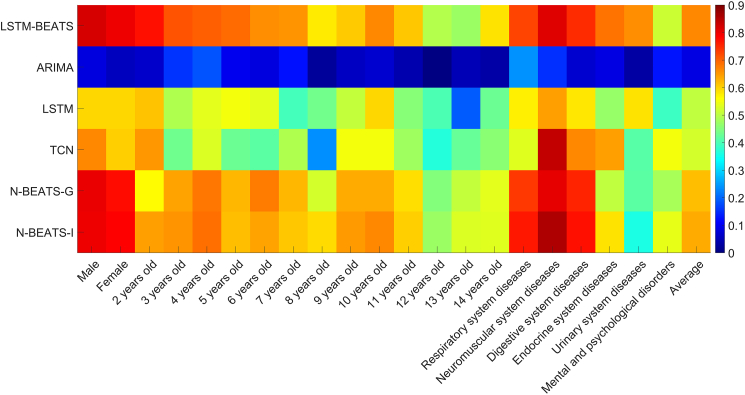
Correlation analysis of features predicted by various models

We presented the correlation analysis between the predicted results and true data for different genders, ages, and system diseases, as illustrated in Figure 2. The predicted correlation coefficients for gender variables exceeded 0.8, while predictions for neuromuscular system diseases (17th column) were particularly accurate. In contrast, predictions for psychiatric disorders were less precise. The correlation coefficients for age groups were maintained at around 0.65. The predictive performance varied across models for different variables. Both versions of the N-BEATS model showed performance close to our model, but overall, our model slightly outperformed the N-BEATS series and was significantly better than the traditional models, ARIMA and TCN. The impetus behind all our work is to address potential future disease outbreaks, resource allocation, and long-term planning issues. Therefore, it is imperative that we return to the analysis of the predicted data points to ensure the value of our work. The utilization of pediatric predictive data by health organizations is crucial for devising strategies. Our work can assist in tackling challenges in the allocation and management of medical resources. By predicting patient visits, we can better plan the resources of pediatric departments, ensuring sufficient medical services are available when needed, and efficiently allocate manpower, supplies, and equipment.

**Figure 3:**
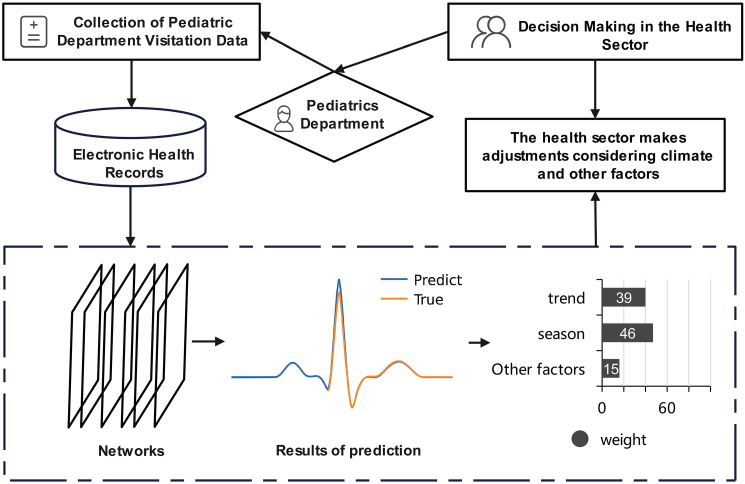
The flowchart of our general deep learning framework for the automated prediction of pediatric patient volumes

The workflow of our project is as follows (Figure 3): it begins with data collection and cleaning, followed by the selection of relevant features and the construction of an appropriate predictive model. Subsequently, the model is trained and evaluated while parameters are continuously optimized throughout the process. In the final stages, the predicted results are visualized and applied to true business scenarios. For instance, when predicting an increase in patient visits during peak periods, adjustments can be made to the allocation of medical resources, extending working hours, and ensuring an adequate supply of medication and medical equipment in advance to effectively meet patient demands.

**Figure 4:**
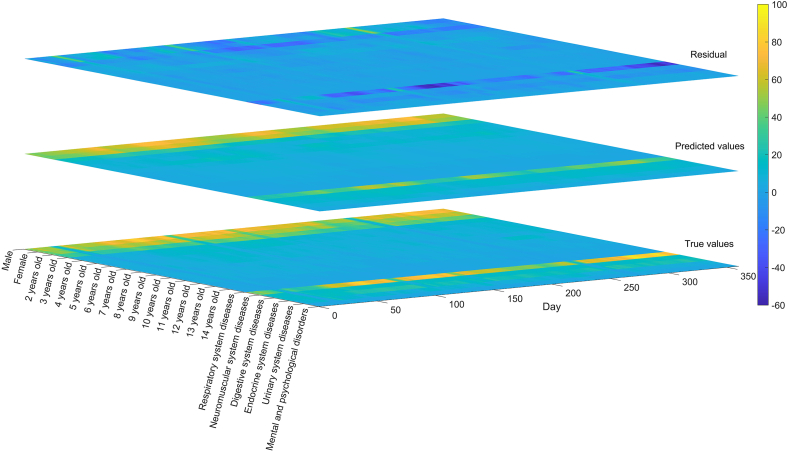
Performance of our LSTM-BEATS model in predicting 21 indicators for the year 2023

**Figure 5:**
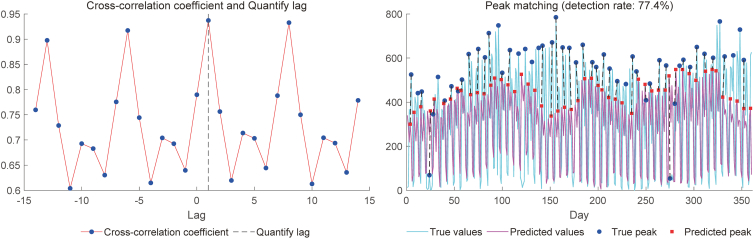
Quantified Lag and Peak Error experiments based on the summed values of all 21 indicators

Figure 4 compares the predicted values of 21 indicators with the true data for the year 2023. It can be observed that certain indicators, such as “3 years old,” “4 years old,” “Respiratory system diseases,” and “Neuromuscular system diseases,” as well as specific time point .g., 2023-01-25, 2023-05-02, 2023-10-05), show some degree of deviation. Nevertheless, the predictions for the majority of indicators fall within an acceptable range. Additionally, we conducted quantified lag and peak error experiments based on the summed values of all 21 indicators, as shown in Figure 5. The results demonstrate that the predictions effectively capture the overall trend variations of the actual data. Figure S1 illustrates the prediction results of LSTM-BEATS for 21 single-variable indicators (including gender, age, and disease categories) in terms of RMSE, MASE, and correlation coefficient.

**Figure 6:**
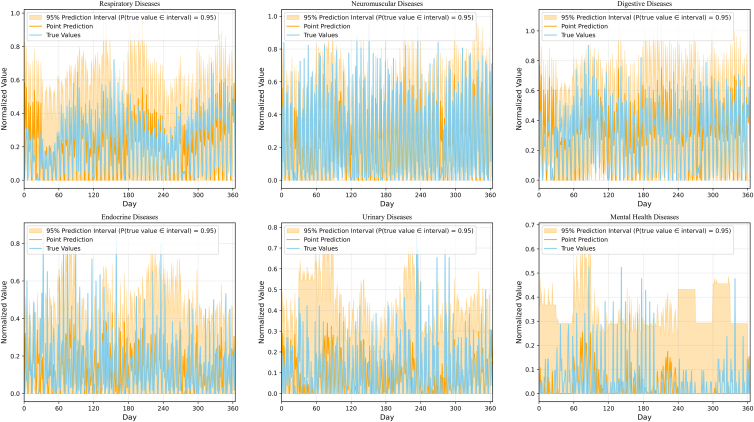
Prediction interval estimation based on conformal prediction The x axis denotes the day index, and the y axis shows the normalized incidence (patient volume). The orange shaded band represents the 95% adaptive prediction interval, whose width is dynamically adjusted according to seasonal regimes: 1.5× during peak periods, 1.0× during normal periods, and 0.8× during off-peak periods. The orange line indicates the model’s point prediction, while the blue line corresponds to the observed values.

To further evaluate the reliability and practical utility of the proposed predicting model, we incorporate prediction interval estimation based on conformal prediction^27^ and assess its empirical coverage using data from 2023 (Figure 6). For each target series, we construct 95% prediction intervals, enabling uncertainty quantification alongside point prediction. The results indicate that for respiratory diseases, the observed values are almost entirely contained within the corresponding 95% conformal prediction intervals, suggesting good calibration and high empirical coverage. This performance is largely attributable to the pronounced seasonality and periodicity of respiratory diseases. Beyond supporting the accuracy of point predictions, the calibrated intervals provide reliable uncertainty bounds that can be directly used to inform operational decisions (e.g., workforce planning and inventory buffers), which is particularly relevant given that respiratory diseases constitute a major share of pediatric visits in our setting. For the remaining five disease systems (neuromuscular, digestive, endocrine, urinary, and mental diseases), we observe heterogeneity in interval performance, with a small number of observations falling outside the 95% intervals. These deviations are mainly associated with abrupt changes and anomalous fluctuations; for instance, mental and psychological disorders exhibit weaker periodicity, making uncertainty more difficult to capture using seasonal patterns alone. Overall, conformal prediction intervals appear robust under typical conditions, while enhancing responsiveness to sudden regime shifts and extreme events remains an important direction for future work.

**Figure:**
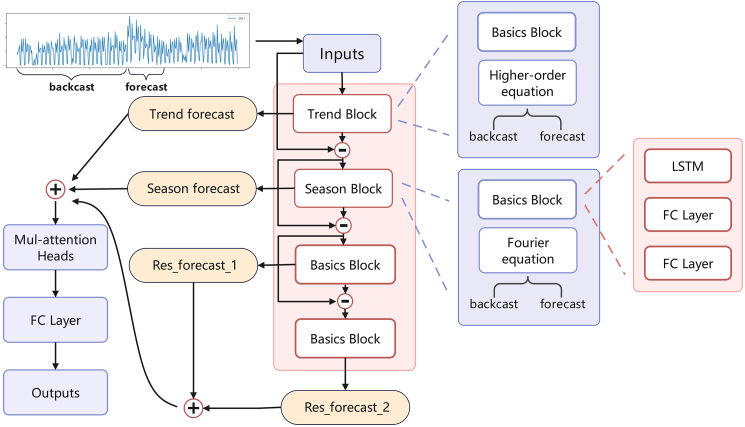
LSTM-BEATS structure

**Figure:**
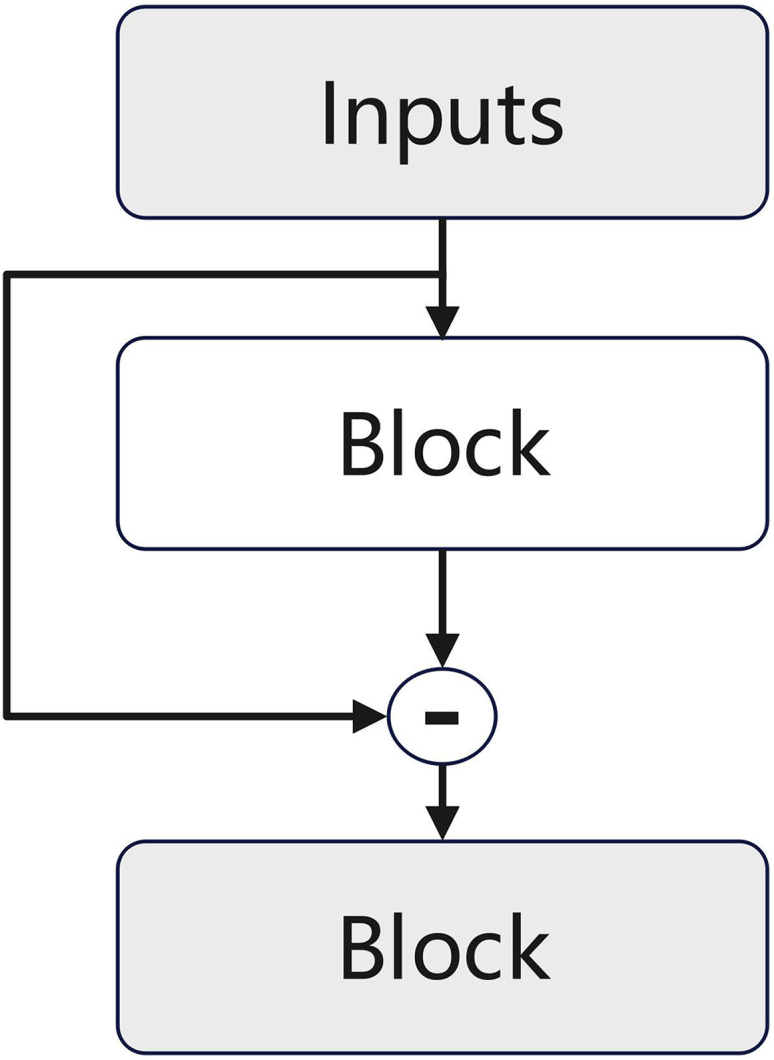
The structure of hierarchical dual-residual

We displayed the prediction effectiveness under different modes and various step ratios, as listed in Table 2. Rolling prediction utilizes only the known historical data for each prediction and updates the model to include new observational data after each prediction. Recursive prediction uses the results of the previous prediction as input for the next step, conducting predictions recursively. We compared the predicting outcomes of both modes to address the needs of different real-world scenarios. The results in the table show that rolling prediction significantly outperforms recursive prediction. This is attributed to the fact that recursive prediction accumulates errors, as each new prediction is built upon the potentially erroneous predictions made previously. As the ratio of observed data to predicted data steps increases, the model’s predictive performance gradually improves, aligning with real-world scenarios where longer periods of observation enable more accurate predictions. Through multiple experiments, the optimal ratio has been determined to be 7:1, as listed in Table 3. This is because a ratio that is too high can lead to a significant reduction in the sample size of data, resulting in decreased model fitting effectiveness. Conversely, a ratio that is too low can lead to a shorter retrospective data length, capturing fewer features and thus affecting predictive performance. In summary, rolling prediction places greater emphasis on using the most up-to-date data to update the model, making it suitable for capturing rapidly changing trends. In scenarios, where historical data are updated in a timely manner, rolling prediction is more appropriate for short-term prediction. On the other hand, recursive prediction is used when there is no timely historical data to refer back to for predicting longer-term scenarios in the future. However, it is important to be mindful of the issue of cumulative errors and to adjust the relevant parameters accordingly.

**Table 2:** Prediction performance across different proportions and prediction methods

Prediction mode	Step ratio	RMSE	MASE	Average correlation coefficient
Rolling Prediction	3:1	0.141	0.663	0.610
	5:1	0.132	0.611	0.626
	7:1	0.130	0.610	0.676
Recursive Prediction	3:1	0.154	0.678	0.599
	5:1	0.153	0.664	0.600
	7:1	0.144	0.663	0.609

MASE, mean absolute scaled error; RMSE, root-mean-square error.

**Table 3:** Definitions of input window, prediction range, stride, and step ratio (ratio = 7:1)

Basic time step	Time step ratio	Backcast time step (Inputs)	Predicted time step (Outputs)	Feature
30	7:1	210	30	21

**Table 4:** Pediatric patient count and distribution statistics, 2017-2023

Date	Male	…	Age of 6	…	Respiratory System	…
2017.01.02	45	…	10	…	50	…
2017.01.03	38	…	25	…	18	…
…	…	…	…	…	…	…
2023.12.29	95	…	20	…	115	…

**Table 5:** The primary network layer parameters

	Description	Input dimension	Output dimension
Inputs layers	–	(64, 21, 210)	–
Trend Block layers	LSTM	(64, 21,210)	(64, 21,256)
	FC Layer	(64, 21, 256)	(64, 21, 128)
	FC Layer	(64, 21, 128)	(64, 21, 64)
	Dropout Layer	(64, 21, 64)	(64, 21, 64)
	Higher-order equation	(64, 21, 64)	(64, 21, 210)
Season Block layers	LSTM	(64, 21,210)	(64, 21,256)
	FC Layer	(64, 21, 256)	(64, 21, 128)
	FC Layer	(64, 21, 128)	(64, 21, 64)
	Dropout Layer	(64, 21, 64)	(64, 21, 64)
	Fourier equation	(64, 21, 64)	(64, 21, 210)
Basic Block_1 layers	LSTM	(64, 21, 210)	(64, 21, 256)
	FC Layer	(64, 21, 256)	(64, 21, 128)
	FC Layer	(64, 21, 128)	(64, 21, 30)
Basic Block_2 layers	LSTM	(64, 21, 30)	(64, 21, 256)
	FC Layer	(64, 21, 256)	(64, 21, 128)
	FC Layer	(64, 21, 128)	(64, 21, 30)
Attention layers	–	(64, 21, 30)	(64, 21, 30)
FC layers	–	(64, 21, 30)	(64, 21, 30)
Output layers	–	(64, 21, 30)	–

**Table 6:** Detailed hardware and software configuration of the experimental environment

Configuration	Version
Operating System	Ubuntu 18.04.6
Memory	DDR4 32G
CPU	Intel Xeon Silver 4210R 2.40GHZ
GPU	NVIDIA GeForce GTX 3080T1,12G
CUDA	11.8
cuDNN	7.6.5
Python	3.8.11
TensorFlow	2.10.0

## Discussion

Long-term disease prediction can predict the peak or severity of the upcoming influenza season,^28^ and hospitals can utilize short-term predictions to anticipate the caseload for the next week or month.^29^ Particularly for pediatric departments, children are more susceptible to infections during the influenza season; hence, effective resource planning is crucial for safeguarding their health. Moreover, it can assist public health organizations in making informed decisions during the influenza season. By having advanced knowledge of the predicted influenza peak, health departments can take timely measures, such as promoting vaccination, enhancing educational outreach, and instituting stricter influenza control measures. These initiatives can help reduce the number of influenza cases, slow the spread of the epidemic, and thereby protect the health of the entire community.

Our proposed LSTM-BEATS model integrates a trend module, a seasonal periodic module, and a residual module, and introduces a multi-head self-attention mechanism. It is adept at capturing trends and seasonal variations. Taking the respiratory disease system as an example, we provided the high baseline level in 2022 (Figure S2 and Table S2). The 2022 data likely included a substantial number of COVID-19 (novel coronavirus pneumonia) cases and their related complications (such as pneumonia). The surge in infections directly led to an abnormal increase in patient visits for respiratory diseases. In 2023, patient visits decreased by over 25% year-over-year each quarter, which is clinically attributed to the formation of an immune barrier by 2023. With large-scale natural infections and vaccinations, the population has established a stronger immune barrier. This has significantly reduced the infection and severe case rates of the virus. Even when infections occur, symptoms tend to be milder, and many patients with mild symptoms may no longer seek hospital treatment.

According to the analysis of the model’s predictions and actual patient visit data (Table S3), the incidence rate for children aged 0–6 is significantly higher than that for children aged 7–14. This disparity is likely closely related to the rapid growth and development occurring during the 0–6 age stage. During this period, children’s immune systems are not fully developed; their levels of antibodies and overall immune mechanisms are less robust compared to older children, leaving them more vulnerable to various infectious diseases. Furthermore, children in this age group are frequently exposed to a wider range of new environments as they begin attending daycare, kindergarten, or engaging in outdoor activities. These factors considerably increase their chances of contracting contagious illnesses. Notably, during flu seasons and outbreaks of local infectious diseases, the risk of illness in children aged 0–6 becomes even more pronounced.

From Table S3, an analysis of the seasonal distribution of patient visits reveals that the number of visits in the second, third, and fourth quarters is significantly higher than in the first quarter. This phenomenon is likely linked to the geographical location of the Chaoshan region, which lies along the Tropic of Cancer. Chaoshan is characterized by a subtropical climate with high temperatures, abundant rainfall, and high humidity, particularly during the summer and autumn seasons. Such warm and humid conditions create an ideal breeding ground for many pathogens, including those causing bacterial dysentery, viral gastroenteritis, and seasonal influenza. These factors collectively contribute to the higher incidence of regional and seasonal diseases in these quarters. Moreover, the hot and humid weather in summer can exacerbate hygiene concerns, where poor dietary and personal hygiene habits may lead to a surge in gastrointestinal diseases. In contrast, the first quarter typically corresponds to the colder winter months. While winter is also a peak period for flu outbreaks, outdoor activities tend to be less frequent, and the colder temperatures restrict certain transmission pathways of pathogens. As a result, disease incidence and patient visits are relatively lower during this quarter. However, as winter transitions to spring and temperatures gradually rise, the second quarter often sees an increase in various diseases, leading to a surge in patient visits. These findings highlight not only the impact of seasonal changes on disease transmission but also the significance of regional geographical and climatic factors in shaping the distribution of high-incidence periods for diseases.

In this study, we incorporated the predicted results from 2017 to 2023 (Figure S3). In this figure, the right side of the graph, marked with a black line, represents predictions for the 2023 test set. This visual representation effectively demonstrates the robustness and applicability of our approach over an extensive period spanning 2,555 days across years. By including this setup, we aim to validate the adaptability and stability of the deep learning model when handling long time-series data.

In practice, the predicted results of this study demonstrate tangible value for optimizing workforce allocation and resource planning during peak periods of pediatric healthcare demand in the Chaoshan region. Accurate prediction of pediatric outpatient and emergency visits is essential for shifting healthcare resource management from a predominantly “reactive” mode to a more “proactive” and anticipatory framework. The potential clinical and operational benefits are reflected in two main aspects.

First, human resource allocation can be more flexible and efficiently managed. By identifying high-demand periods—such as the influenza season from September to November—a flexible staffing strategy can be adopted. During non-peak periods, routine demand can typically be met by one to two staff members, whereas peak periods require an expansion to three to four staff members, corresponding to an approximately 80–100% increase in staffing capacity. Such adjustments can substantially reduce patient waiting times, mitigate staff fatigue associated with sudden workload surges, and help maintain stable and safe clinical operations during periods of elevated demand.

Second, drug and medical supply planning can be better aligned with anticipated demand. Predictions of patient volume and disease composition enable more precise planning of essential medications and consumables (e.g., oseltamivir). A demand-driven, dynamically adjusted procurement and inventory strategy helps ensure adequate supply during peak influenza seasons, reduces the risk of stockouts, and minimizes financial losses associated with overstocking or medication expiration.

Although this study primarily evaluates model performance using predictive accuracy metrics and statistical significance tests, it is important to emphasize that improvements in statistical indicators do not automatically translate into clinical or operational utility. The real-world value of visit prediction critically depends on how predictions are embedded within concrete decision-making workflows. Accordingly, we distinguish two practical application pathways with distinct decision objectives and interpretation boundaries. Total-visit prediction primarily supports hospital- or department-level capacity and operations planning, including staffing allocation, clinic-room scheduling, and coordination of ancillary services (e.g., triage and laboratory capacity), with the aim of reducing congestion and workforce mismatch during peak periods. In contrast, disease- or syndrome-specific prediction is more closely aligned with public health surveillance and specialty preparedness, supporting targeted staffing buffers and medication or supply planning during high-risk seasons such as respiratory infection outbreaks. In this context, the value of predicting extends beyond reducing a single error metric, enabling earlier detection of shifts in disease composition and peak-risk windows, and thereby facilitating more timely and tailored preparedness actions.

We further emphasize that predicting interpretation must be contextualized by external epidemiological drivers and behavioral changes to avoid over-interpretation of model outputs. For example, the observed decline in influenza-related visit rates in the fourth quarter of 2024—particularly among younger age groups—may plausibly reflect the combined effects of strengthened vaccine-mediated immunity and the persistence of post-COVID preventive behaviors, such as mask use, improved hand hygiene, household isolation practices, and school-level non-pharmaceutical interventions. Age-stratified differences may additionally be explained by variation in exposure controllability, with preschool children typically operating within more confined and supervised environments, as well as differences in family-level protective investments. Importantly, a reduction in visit rates does not necessarily imply a proportional reduction in disease incidence, as healthcare-seeking behavior and increased home management of mild cases may also contribute to this pattern. Consequently, the proposed predicting framework should be regarded primarily as a demand-side management tool, supporting early signals of impending resource pressure and enabling flexible operational responses, rather than as a direct substitute for disease burden estimation or causal inference.

Overall, the contribution of this work extends beyond improved predictive accuracy. It provides a predictive interface that can be aligned with decision-making at multiple levels—using total-demand predictions for capacity scheduling and disease-specific predictions for structural risk surveillance. Future work will focus on evaluating the real-world impact of prediction-informed workflows, such as staffing adjustments and inventory interventions, on operational outcomes, including waiting times, congestion, understaffing, and stockouts, thereby strengthening the evidence base for practical deployability.

### Limitations of the study

The present study is subject to several limitations. First, the model was developed and validated using pediatric influenza case data from a single hospital in one region, which may limit its generalizability to other populations or epidemiological settings. Second, the current framework is based on aggregated time series data and does not explicitly incorporate individual-level clinical or behavioral information, which may affect disease dynamics. Third, while abrupt change points were included to improve predictive performance, the specific epidemiological or external factors underlying these changes were not explicitly analyzed.

## Resource availability

### Lead contact

Further information and requests for resources should be directed to and will be fulfilled by the lead contact, Qiu-Ling Tang (tql622@163.com).

### Materials availability

This study did not generate new unique reagents.

### Data and code availability

• Data: The original data reported in this article cannot be deposited in a public repository due to patient privacy and ethical restrictions. Contact the lead contact to request access.
• Codes: The codes of benchmark models (ARIMA, N-BEATS-G, N-BEATS-I, LSTM, TCN, Prophet), which were implemented using Darts, can be found at https://github.com/unit8co/darts [arXiv: 2110.03224]. (key resources table). The codes of the LSTM-BEATS model can be found at https://github.com/WangSiQi1202/Pediatrics-DeepLearning/tree/Deep_learning [zenodo: 18598956]. (key resources table).
• Other items: Any additional information required to reanalyze the data reported in this article is available from the lead contact upon request.

## Acknowledgments

We thank the following funding sources for their support: Strategic Priority Research Program of 10.13039/501100002367Chinese Academy of Sciences (Grant/Award Numbers: XDB38000000), the Medical Scientific Research Foundation of Guangdong Province (Grant/Award Numbers: B2025236), the Medical Health Science and Technology Project of Shantou (Grant/Award Number: 240428166497960), the Shantou Science and Technology Program Healthcare Category Project (Grant/Award Number: 221122176494982), and the Guangdong Medical Association Clinical Research Fund - Special Program for Chronic Disease Prevention and Cardiovascular and Cerebrovascular Health (Grant/Award Number: 2025MB-A1002).

## Author contributions

Conceptualization, S.-Q.W., J.-L.F., Y.-L.C, H.C., X.-L.Z., H.-W.W., and Q.-L.T.; data curation, J.-L.F., Y.-X.Y., and S.-X.L.; formal analysis, J.-L.F. and X.-L.Z.; funding acquisition, X.-L.Z., H.-W.W., and Q.-L.T.; investigation, J.-L.F., H.C., Y.-W.C., X.-L.Z., and H.-W.W.; methodology, S.-Q.W. and X.-L.Z.; project administration, Q.-L.T.; software: S.-Q.W. and Y.-L.C.; supervision, Y.-W.C., X.-L.Z., and H.-W.W.; validation, H.C, Q.-L.T.; visualization, S.-Q.W., J.-L.F., Y.-L.C., and X.-L.Z.; writing – original draft: S.-Q.W., J.-L.F., and Y.-L.C.; writing – review and editing, H.C., X.-L.Z., and Q.-L.T.

## Declaration of interests

The authors declare no competing interests.

## STAR★Methods

### Key resources table

**Table:** 

REAGENT or RESOURCE	SOURCE	IDENTIFIER
**Software and algorithms**		

TensorFlow v2.10.0	TensorFlow official website	arXiv:1605.08695
Darts v0.28.0	Journal of Machine Learning Research	arXiv: 2110.03224
LSTM-BEATS	Zenodo	zenodo: 18598956

### Experimental model and study participant details

#### Study population

Institutional Review Board approval was obtained. This study was approved by the Ethics Committee of the Second Affiliated Hospital of Shantou University Medical College (2023-48), which waived the requirement for written informed consent owing to the use of de-identified retrospective data. We have collected EHRs of pediatric outpatients and emergency department of the Second Affiliated Hospital of Shantou University Medical College, the largest children’s medical center in Chaoshan region. The pediatric patients at this center predominantly come from the surrounding Chaoshan region. Due to the high volume of visits and the wide variety of diseases, the pediatric patient profile at this center to some extent reflects the general characteristics of pediatric visits in the Chaoshan area.

This study selected pediatric outpatient and emergency visit data from Shantou University Medical College Second Affiliated Hospital between 2017 and 2023. The inclusion criteria for the search were based on the time of visit, gender, age, residential address, and disease diagnosis. The diagnoses were categorized according to the International Classification of Diseases (ICD-10). We ultimately chose the five most common system-based diseases for analysis: respiratory system diseases, neuromuscular system diseases, digestive system diseases, endocrine system diseases, urinary system diseases, as well as the increasingly prevalent mental and psychological disorders. These six disease categories were included in the analysis. The inclusion criteria were children aged 0–14 years with a clear diagnosis, and a total of 174,953 pediatric patients were included in the study. Among them, 56.99% were male and 43.01% were female. The age distribution of the subjects ranged from 0 to 14 years, with a median age of 6.0 years and a 95% confidence interval ranging from 2.0 years to 13.0 years. After data cleaning and processing, we obtained a dataset comprising 2,555 samples (i.e., 2,555 consecutive days), covering a span of six years and including 278,506 medical records diagnosed with common system diseases in this retrospective study. The dataset comprises 22 columns of information (including the date and 21 indicators), providing a comprehensive record of the daily number of pediatric patients diagnosed with common system diseases. The data includes details such as the date, age distribution of patients, and types of disease systems, which will be used as numerical inputs for the model. The model will predict a corresponding set of 22 numerical values, representing the patient count. For specific information, as detailed in below table. It should be noted that the same child may have multiple visits, resulting in the number of visit records exceeding the actual number of patients. Consequently, we observe the situation where there are “a total of 278,506 medical records for common system diseases, while the true number of pediatric patients is 174,953.”

### Method details

#### Deep learning model

Deep learning represents an advancement in artificial neural networks, characterized by multiple layers of representation, each creating non-linear transformations. It enables the rapid discovery of complex relationships within large datasets and performs tasks such as prediction and classification by simulating these complex relationships.^30^ A primary advantage of deep learning models is their ability to automatically learn features without the need for manual feature selection, which distinguishes them from other methods. The present study proposes a pediatric patient volume prediction model capable of multivariate concurrent predicting and names the model as “LSTM-BEATS” below figure.

This model utilizes the advantage of LSTM to capture long-term context dependence and combines with the fully connected layer as the foundational module to realize the trend module, season module and residual module. Additionally, the model is endowed with a multi-head attention mechanism to apportion weights across the various modules, thereby enhancing its predictive accuracy and interpretability. The predictive model ingests observational data as inputs and processes it through the trend module to capture the underlying trends, which are then outputted as one part of the result. Concurrently, the residuals from the initial data minus the trend output are channeled into the seasonal module. After the seasonal module identifies the seasonal patterns, the remaining residuals are fed into the subsequent module. The final module, based on the foundational module, aims to capture the impact of abrupt changes. The outputs from the three modules are then passed to a multi-head attention mechanism to assign different weights to each. Ultimately, the model produces the predicted outcomes for future variables. This architectural approach significantly augments the interpretability of the deep learning model, which is conducive to the meticulous observation and analysis of predictive outcomes. It aids health departments in analyzing the impact of various factors, thereby facilitating a more strategic and robust response to the exigencies of disease surges and unforeseen circumstances.

##### Basic block

The basic block of LSTM-BEATS consists of a 256-unit LSTM (Long Short-Term Memory),^31^ followed by two fully connected layers with 128 and 64 neurons, respectively. LSTM networks are capable of maintaining sequence data over extended periods, addressing the issue of vanishing gradients, and employing gating mechanisms to capture long-term dependencies. The Long Short-Term Memory (LSTM) utilizes a gating mechanism that incorporates an input gate, a forget gate, and an output gate, which can be mathematically represented as Equations 1, 2, 3, 4, 5, and 6. When the gates are closed, they prevent the alteration of current information, thereby allowing the network to understand the dependencies from prior information. Conversely, when the gates are open, they do not entirely overwrite the previous information but instead perform a weighted average between the previous and current information. Consequently, regardless of the depth of the network or the length of the input sequence, as long as the gates are open, the network retains this input information.

(1) $$h_{t}=f\left(\begin{array}{c} W_{h} \end{array}\cdot x_{t}+U_{t}\cdot h_{t-1}+b_{h}\right)$$

Where *W*_*h*_ and *U*_*t*_ are weight matrices, *b*_*h*_ is the bias term, *f*(*x*) is a non-linear function, typically chosen as *tanh*, and *h*_*t*_ represents the regular hidden state.

(2) *f*_*t*_ = *σ*(*W*_*f*_·[*h*_*t*-1_,*x*_*t*_]+*b*_*f*_)

(3) *i*_*t*_ = *σ*(*W*_*i*_·*x*_*t*_+*U*_*t*_·*h*_*t*-1_+*b*_*i*_)

(4) *o*_*t*_ = *σ*(*W*_*o*_·*x*_*t*_+*U*_*o*_·*h*_*t*-1_+*b*_*o*_)

(5) *c*_*t*_ = *f*_*t*_∗*c*_*t*-1_+*i*_*t*_∗*tanh*(*W*_*c*_·*x*_*t*_+*U*_*c*_·*h*_*t*-1_+*b*_*c*_)

(6) *h*_*t*_ = *o*_*t*_∗*tanh*(*c*_*t*_)

Where *i*_*t*_ is the input gate, *f*_*t*_ is the forget gate, *σ* is the sigmoid function, *c*_*t*_ is the memory cell, which is updated using the inputs from the input and forget gates. After updating the memory cell, the output gate is used to determine the output of the LSTM unit, denoted as *o*_*t*_. Finally, the *c*_*t*_ is processed through the output gate and through the *tanh* function to obtain the final confirmed output content.

The fully connected layer^32^ is a fundamental layer structure in deep learning neural networks. Within this layer, each neuron is connected to every neuron in the preceding layer, with each connection possessing a weight parameter. Consequently, the output of the fully connected layer is a weighted sum of all neurons from the preceding layer, as depicted in Equation 7. Such a configuration enables the fully connected layer to learn complex nonlinear relationships within the input data, thereby accommodating various types of data.

(7) *Y* = *X*×*W*^*T*^ + *b*

Let *Y* denotes the output, *X* denotes the input, *W*^*T*^ denotes the transpose of the weight matrix, and *b* denotes the bias.

##### Trend module

The module is inspired by the N-BEATS^33^ model, which was originally designed to address point predicting for univariate time series. The N-BEATS model employs only fully connected networks to achieve time series prediction, offering interpretability and can be approximated by practitioners using a “seasonality-trend-level” decomposition approach. Fully connected layers exhibit limited capacity for modeling sequential data and fail to capture long-term dependencies. Moreover, when the input feature dimensions are high, the number of parameters increases rapidly, which can escalate the model complexity. Without proper regularization and model selection strategies, this may lead to overfitting issues. Therefore, based on this foundation, we have reduced the number of fully connected layers by seven and have incorporated LSTM networks, leveraging memory cells to detect and retain long-term dependencies. The gating mechanism enables parameter sharing, which in turn reduces the number of parameters that require training, aiding in the reduction of the risk of overfitting. The trend module is composed of a basic block and a polynomial function to model the trend variations, which can be represented in matrix form as follows:

(8) $${\hat{y}}_{l}^{tr}=T\theta_{l}^{f}$$

Here, *l* denotes the output of the basic block, *T* = [1,*t*, …,*t*_*p*_] is the power matrix of *t,* and *p* can be set to a relatively small value. In this study, *p* is set to 3. This polynomial fits the trend variations through a higher-order function.

##### Seasonal periodic module

The regularity of seasonal variations is typically represented using periodic functions. Fourier series can fit periodic data as a linear combination of sine and cosine functions. By increasing or decreasing the number of sine and cosine terms, the complexity of the fit can be flexibly adjusted to accommodate seasonal variations of different types and complexities. The matrix form used can be represented as follows:

(9) $${\hat{y}}_{l}^{seas}=S\theta_{l}^{f}$$

*S* = [1,*cos*(2*πt*), …,*cos*(2*πit*)),*sin*(2*πt*), …,*sin*(2*πit*))], where *i* is set to 3 in this study.

Additionally, the input to the seasonal module is the residual part of the trend module, that is, the trend module retains the correct parts as the trend output, and the parts that differ from the original input and the trend result are output as residuals, serving as the input for the next module. In this case, it serves as the input for the seasonal module to learn periodic patterns.

##### Residual module

The concept of “residuals” was initially introduced by He et al.^34^ in an innovative Convolutional Neural Network (CNN) architecture, where the input of the layer stack is added to the result before being passed to the next layer. This method addresses the vanishing gradient problem in deep networks, enabling the network to be trained at a greater depth. However, it lacks interpretability. In contrast, Oreshkin et al.^33^ proposed a hierarchical dual-residual structure below figure, which eliminates parts that can be successfully approximated and retains them, leaving the parts that are not successfully fitted to be learned by the next module as input. This can be represented as follows:

(10) $$x_{l}=x_{l-1}-{\hat{x}}_{l-1},\hat{y}=\sum_{l}{\hat{y}}_{l}$$

In this study, the residual part of the trend module serves as the input for the seasonal module to learn periodic patterns. Similarly, the residual part of the seasonal module is fed as input to the next module to learn the characteristics of abrupt change points. To address the outbreak infections caused by the control of new virus sources by health organizations, our method not only includes trend and seasonal modules that capture structural changes but also retains residual parts to capture the occurrence of abrupt change points. This approach overcomes the difficulty of traditional methods that cannot simultaneously consider structural changes and abrupt change points. The output of different modules retains the special characteristics of the module, making the model more interpretable and providing clearer decision-making for real business scenarios.

##### Multi-head self-attention mechanism

The multi-head self-attention mechanism is a crucial component of the Transformer,^35^ and its application significantly enhances the capabilities of the self-attention layer. This mechanism enables the model to learn diverse attention representations, where each head can focus on different relationships or features within the sequence, capturing richer semantic information. It also allows the model to interact and transmit information directly, thereby better understanding the relationships between different elements within the sequence. The multi-head attention mechanism introduces a series of differentiated query (Q), key (K), and value (V) matrix combinations, which are randomly generated during model initialization and subsequently optimized through training. This allows the input vectors to be mapped into multiple distinct representational subspaces. Such a design enables the model to independently capture and focus on multiple positions within the sequence across different subspaces. Consequently, when synthesizing the outputs of all heads, a comprehensive representation enriched with diverse positional information is obtained. In this way, the multi-head attention mechanism provides the model with the ability to capture information in parallel across different representational spaces, greatly enriching the model’s understanding and processing capabilities of sequential data.

(11) *MultiHead*(*Q*′,*K*′,*V*′) = *Concat*(*head*_1_,*head*_2_, …,*head*_*h*_)*W*^*o*^

(12) $${head}_{i}=Attention\left(QW_{i}^{Q},KW_{i}^{K},VW_{i}^{V}\right)$$

In the formula, $W_{i}^{Q}\in R^{d_{model}\times d_{q}}$, $W_{i}^{K}\in R^{d_{model}\times d_{k}}$, $W_{i}^{V}\in R^{d_{model}\times d_{v}}$, $W_{i}^{O}\in R^{{hd}_{v}\times d_{model}}$, *h* represents the number of attention heads. Under the multi-head attention mechanism, each set of attention maintains its own input and output weight matrices.

In this study, the trend module, seasonal periodic module, and the residual module serve as inputs to the multi-head self-attention mechanism. After training and optimization, the model enhances the processing of important information while suppressing the impact of irrelevant or secondary information, thereby improving the interpretability of the model and providing a clearer analytical approach to the prediction results. In real-world scenarios, seasonal changes often bring irregular climatic variations. Children with weaker resistance tend to experience respiratory infections and fever in stages during adverse climate changes. When facing emerging pathogen sources, there is an accompanying gradual increase in the number of infections. Therefore, to address the complex patterns in different scenarios, the adaptive capacity of the predicting model plays a crucial role. The addition of the multi-head self-attention mechanism helps the model to adapt to the varying degrees of different factors, assign different weights to each module, and make interpretable decisions.

#### Evaluation metrics

To validate the effectiveness of LSTM-BEATS, we conducted a performance comparison with various other time series predicting models. We employed RMSE (Root-Mean-Square Error) and MASE (Mean Absolute Scaled Error) as evaluation metrics, complemented by the Pearson correlation coefficient for correlation validation. By comparing these metrics, we can comprehensively assess the model’s performance across different aspects. Such a comparison helps us to identify the strengths and limitations of our model in time series prediction tasks and provides guidance for further improvement.

##### Root-mean-square error (RMSE)

The RMSE is a widely used metric for assessing the error of predictive models. It measures the degree of difference between the model’s predicted values and the actual observed values, providing an intuitive understanding of the model’s predictive accuracy. The mathematical representation is given by:

(13) $$RMSE\left(y,\hat{y}\right)=\sqrt{\frac{1}{n}\sum_{i=1}^{n}{\left(y_{i}-{\hat{y}}_{i}\right)}^{2}}$$

where *n* is the number of observations, *y*_*i*_ is the actual observed value, and ${\hat{y}}_{i}$ is the predicted value.

##### Mean absolute scaled error (MASE)

Unlike other common error metrics, MASE takes into account the seasonality of the data and compares the prediction error with the error of a simple benchmark model. MASE is scale-free with respect to the data,^36^ thus allowing for the comparison of model performance across different datasets. Its mathematical expression is given by:

(14) $$MASE=\frac{1}{T}\sum_{t=1}^{T}\left|\frac{e_{t}}{\frac{1}{T-m}\sum_{t=m+1}^{T}\left|y_{t}-{\hat{y}}_{t}\right|}\right|$$

*T* represents the length of the time series, *m* denotes the length of the seasonal cycle, and *e*_*t*_is the absolute error between the actual observed values *y*_*t*_ and the predicted value ${\hat{y}}_{t}$.

##### Pearson correlation coefficient

The Pearson correlation coefficient is a statistical measure that assesses the strength and direction of the linear relationship between two variables. It quantifies the degree of linear association between the variables, with values ranging from −1 to 1, where 1 indicates a perfect positive correlation, −1 indicates a perfect negative correlation, and 0 indicates no correlation. The formula for calculating the Pearson correlation coefficient is given by:

(15) $$r=\frac{\sum \left(X-\bar{X}\right)\left(Y-\bar{Y}\right)}{\sqrt{\sum {\left(X-\bar{X}\right)}^{2}}\sqrt{\sum {\left(Y-\bar{Y}\right)}^{2}}}$$

where *X* denotes the predicted values, *Y* represents the actual observed values, $\bar{X}$ indicates the mean of the predicted values, and $\bar{Y}$ signifies the mean of the actual observed values.

#### Experimental parameter settings

In our study, we adhered strictly to the principle of controlling variables, ensuring that all models in the comparative experiments were configured with identical training parameter settings. This approach was taken to guarantee the comparability and reliability of the experimental results. Specifically, the initial 80% temporal duration of the first five years serves as the training set, followed by the subsequent 20% temporal segment as the validation set, while the sixth-year observations constitute the test set. To accommodate the model’s input structure, the dataset is segmented into temporal segments of length 30, ensuring compatibility with the sequential processing requirements of the architecture. Critical architectural parameters include the configuration of both trend and seasonal periodicity modules with cyclical periods set to 90 and Fourier coefficients fixed at 3, in addition to primary network layer parameters below table.

The training parameters we employed included the number of training epochs, batch size, learning rate, and the choice of optimizer. Initially, we set the training to proceed for 120 epochs, which is a relatively extensive period conducive to the models learning the characteristics of the dataset and achieving a stable training state. We opted for a batch size of 64 and set the learning rate to 10-4, utilizing the Mean Squared Error (MSE) as the loss function. Additionally, we employed the Adam optimizer for updating the model parameters. Other experiments within this paper involve adjustments to other parameters, such as the ratio of review time steps to prediction time steps, which will be elucidated in the “results” section.

All neural network architectures in this study were implemented within the TensorFlow framework. To fully leverage GPU acceleration capabilities, the experimental environment was configured with TensorFlow GPU support on an Ubuntu operating system within a Python environment. This included the installation of the CUDA Toolkit and cuDNN library to enable efficient GPU-accelerated training. The detailed hardware and software configuration of the experimental environment is summarized in below table.

### Quantification and statistical analysis

Statistical analyses and graphs were performed in Python software (Version 3.8.11). Table 1: Predicted 21 indicators for the test set (i.e., the year 2023) using a predefined ratio of past steps to future steps, set at 7:1, in terms of RMSE, MASE, correlation and DM. as shown in. Table S1: 95% confidence intervals (CIs) for RMSE, MASE, and correlation of Table 1, both at the individual target level and for their macro-averaged values. Figure 2: Correlation analysis of features predicted by various models. Figure 5: Quantified Lag and Peak Error experiments based on the summed values of all 21 indicators. Figure 6: Prediction interval estimation based on conformal prediction. Figure S1: The prediction results of LSTM-BEATS for 21 single-variable indicators (including gender, age, and disease categories) in terms of RMSE, MASE, and correlation coefficient (i.e., the year 2023).
