# Supplementary material for: Toward accurate prediction of pediatric epidemic disease patient volume in the Chaoshan region: A deep learning framework
Source: iScience. 2026 Mar 3;29(4):115211. doi: 10.1016/j.isci.2026.115211 (PMC13019580; doi:10.1016/j.isci.2026.115211)

iScience, Volume *29*

Supplemental information

Toward accurate prediction of pediatric epidemic disease patient volume in the Chaoshan region: A deep learning framework

Siqi Wang, Jinlian Fang, Yulin Chen, Hui Chen, Yaowen Chen, Yangxin Ye, Shixin Lai, Xiaolei Zhang, Hongwu Wang, and Qiuling Tang

Table S1: Comparison of macro-averaged performance metrics using stratified block bootstrap

Model	RMSE (95% CI)	MASE (95% CI)	Average correlation coefficient (95% CI)
ARIMA	0.2276 [0.2179, 0.2497]	1.6698[1.3458, 2.0679]	0.0837 [0.0511, 0.1167]
N-BEATS-G	0.1916[0.1829, 0.2150]	1.3090[1.0303, 1.7272]	0.6190[0.5715, 0.6706]
N-BEATS-I	0.1911[0.1832, 0.2135]	1.2783[1.0209, 1.6770]	0.6207 [0.5777, 0.6754]
LSTM	0.1635 [0.1536, 0.1817]	1.1801[0.7317, 1.8437]	0.6337 [0.5843, 0.6859]
TCN	0.2084[0.1955, 0.2385]	1.3891[1.1163, 1.7568]	0.520[0.5127, 0.6233]
Prophet	0.2753[0.2652, 0.3054]	1.9471[1.5271, 2.4228]	0.0122 [0.0468, 0.0625]
LSTM-BEATS	0.1532 [0.1425, 0.1737]	0.8758[0.7314, 1.1275]	0.6207[0.5788, 0.6711]

Figure S1: **The prediction results of LSTM-BEATS for 21 single-variable indicators (including gender, age, and disease categories) in terms of RMSE, MASE, and correlation coefficient (i.e., the year 2023).**

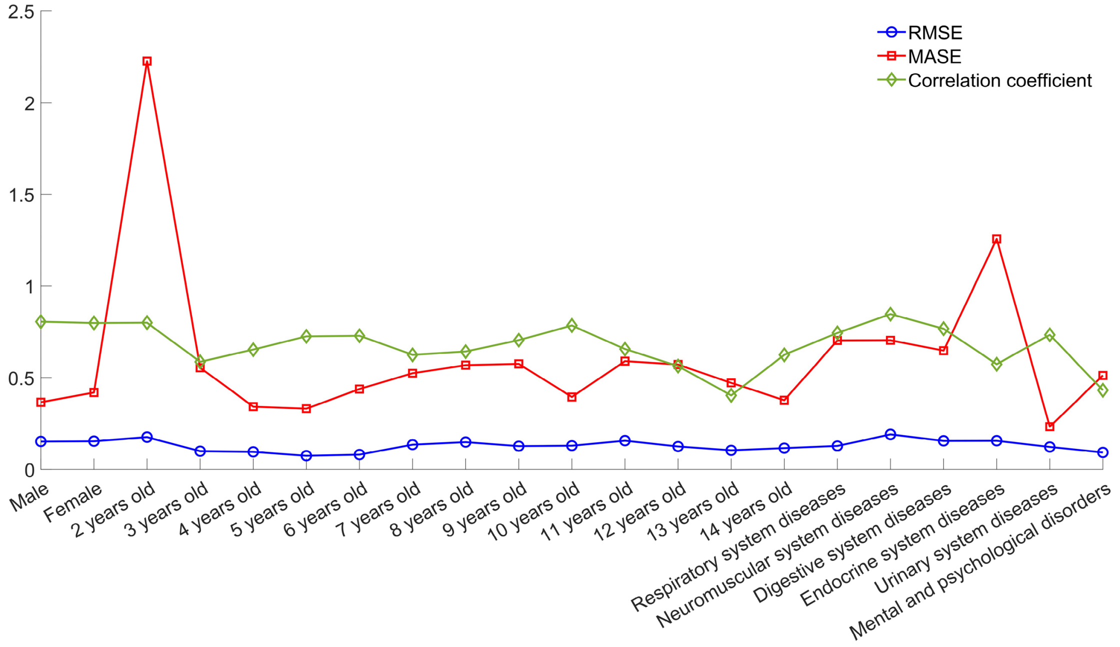

Figure S2: **Quarterly patient visits by disease system (2022-2023) and year-on-year comparative analysis.**

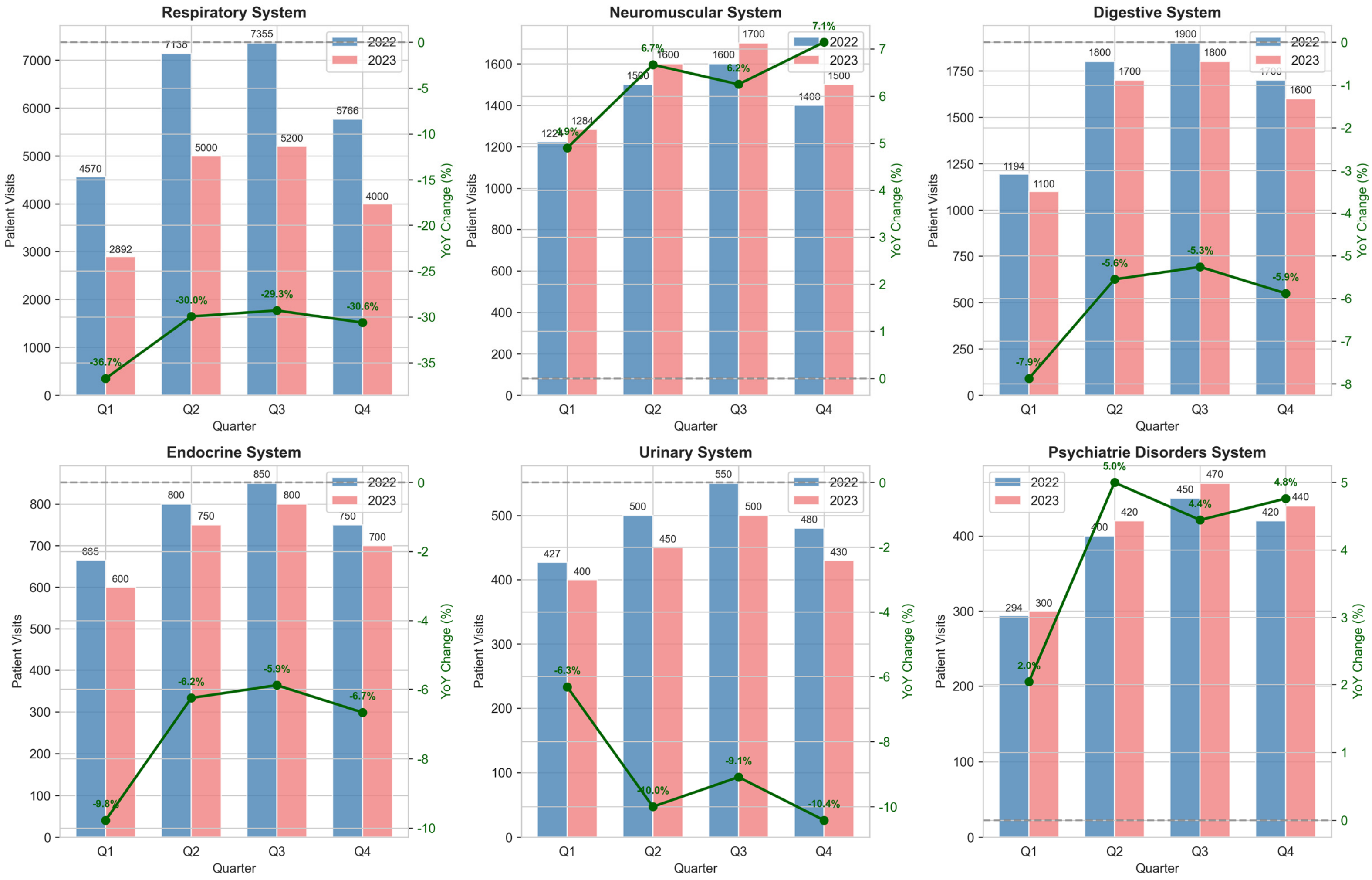

Table S2: Quarterly patient visits by disease system and year-on-year quantitative analysis for 2022-2023

Quarter	Respiratory System	Neuromuscular System	Digestive System	Endocrine System	Urinary System	Psychiatrie Disorders System
**Q1 2022**	4570	1224	1194	665	427	294
**Q1 2023**	2892	1284	1100	600	400	300
**Year-on-year change**	-36.72%	+4.90%	-7.87%	-9.77%	-6.32%	+2.04%
**Q2 2022**	7138	1500	1800	800	500	400
**Q2 2023**	5000	1600	1700	750	450	420
**Year-on-year change**	-29.95%	+6.67%	-5.56%	-6.25%	-10.00%	+5.00%
**Q3 2022**	7355	1600	1900	850	550	450
**Q3 2023**	5200	1700	1800	800	500	470
**Year-on-year change**	-29.30%	+6.25%	-5.26%	-5.88%	-9.09%	+4.44%
**Q4 2022**	5766	1400	1700	750	480	420
**Q4 2023**	4000	1500	1600	700	430	440
**Year-on-year change**	-30.63%	+7.14%	-5.88%	-6.67%	-10.42%	+4.76%

Table S3: **Quarterly patient visit volume by age group in 2023: comparison with predicted results**

	Quarter 1	Quarter 2	Quarter 3	Quarter 4	Average
**Total true value**	9310	10461	10234	10087	10023.00
**Total predicted value**	9750	10984	10746	10585	10516.25
**Error rate**	4.73%	5.00%	5.00%	4.94%	4.92%
**True value (0-6 years old)**	6542	7345	7189	6713	6947.25
**Predicted value (0-6 years old)**	6850	7712	7549	5770	6970.25
**Error rate (0-6 years old)**	4.71%	5.00%	5.01%	-14.05%	0.17%
**True value (7-14 years old)**	2768	3116	3045	3374	3075.75
**Predicted value (7-14 years old)**	2900	3272	3197	4815	3546.00
**Error rate (7-14 years old)**	4.77%	5.01%	4.99%	42.71%	14.37%

Figure S3: The predicted data from 2017 to 2023 shows the patient numbers for various disease systems, with the section to the right of the black line representing the test set predictions for 2023.

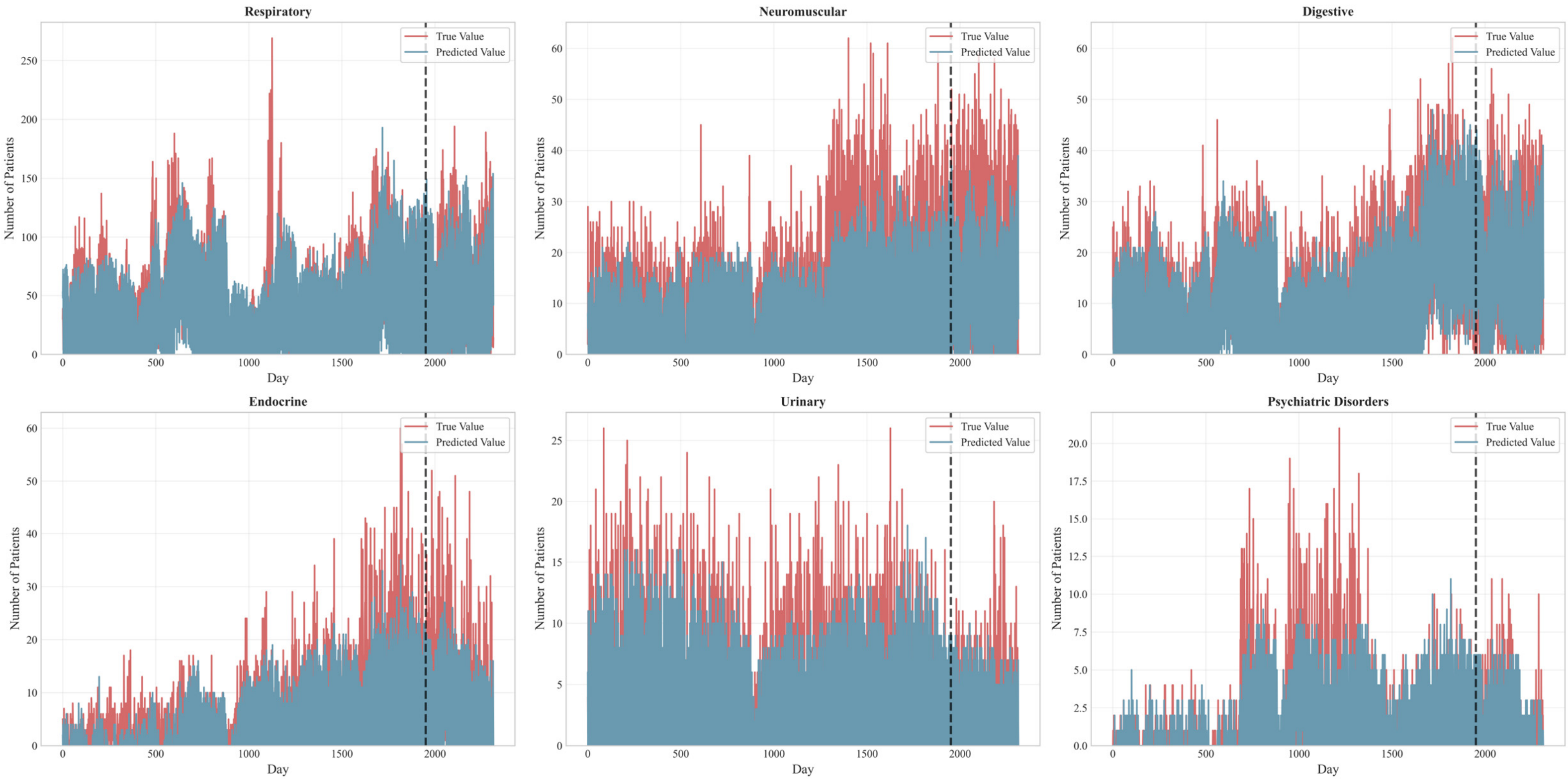

Supplement: Document S1. Figures S1–S3 and Tables S1–S3 [file mmc1.pdf]
